# Harvesting biomechanical energy or carrying batteries? An evaluation method based on a comparison of metabolic power

**DOI:** 10.1186/s12984-015-0023-7

**Published:** 2015-03-20

**Authors:** Eliran Schertzer, Raziel Riemer

**Affiliations:** Department of Industrial Engineering and Management, Ben-Gurion University of the Negev, P.O.B. 653, Beer-Sheva, Israel

**Keywords:** Biomechanical energy harvesting, Battery, Wearable robots, Metabolic rate

## Abstract

**Background:**

Harvesting energy from human motion is an innovative alternative to using batteries as a source of electrical power for portable devices. Yet there are no guidelines as to whether energy harvesting should be preferred over batteries. This paper introduces an approach to determine which source of energy should be preferred. The proposed approach compares the metabolic power while harvesting energy and while using batteries (or any other power supply, e.g., solar panels), which provide equal amount of energy. Energy harvesting is preferred over batteries if the metabolic power required to harvest the energy is lower than that required to carry the batteries. Metabolic power can be experimentally measured. However, for design purposes, it is essential to assess differences in metabolic power as a function of the device parameters.

**The model:**

To this end, based on the proposed approach, we develop a mathematical model that considers the following parameters: the device’s mass, its location on the human body, the electrical power output, cost of harvesting (COH), walking time, and the specific energy of the battery.

**Method:**

We apply the model in two ways. First, we conduct case studies to examine current ankle, knee, and back energy harvesting devices, and assess the walking times that would make these devices preferable over batteries. Second, we conduct a design scenarios analysis, which examines future device developments.

**Results:**

The case studies reveal that to be preferred over batteries, current harvesting devices located on the ankle, knee, or back would require walking for 227 hours, 98 hours, or 260 hours, respectively. This would replace batteries weighing 6.81 kg (ankle), 5.88 kg (knee), or 2.6 kg (back). The design scenarios analysis suggests that for harvesting devices to be beneficial with less than 25 walking hours, future development should focus on light harvesting devices (less than 0.2 kg) with low COH (equal or lower than 0). Finally, a comparison with portable commercial solar panels reveals that under ideal sun exposure conditions, solar panels outperform the current harvesting devices.

**Conclusions:**

Our model offers a tool for assessing the performance of energy harvesting devices.

**Electronic supplementary material:**

The online version of this article (doi:10.1186/s12984-015-0023-7) contains supplementary material, which is available to authorized users.

## Introduction

Consider the following scenario:

*An individual who is planning a three-week hike has the choice of selecting either a biomechanical energy harvester or batteries to provide the energy requirements for all of his/her electronics equipment (camera, cell phone, etc.). What would be the right choice for this hiker?*

Biomechanical energy harvesting from human motion is a green alternative for powering portable electronic devices such as global positioning systems (GPS), mobile phones, and laptop computers. In the past few years a variety of biomechanical energy harvesting devices have been developed, such as the mechanical flashlight, as well as harvesters located on the ankle, in/on the shoes [1-4], on the knees [5,6], or on the back [7,8]. Such devices can supply electrical energy to users who do not have direct access to an electric grid for long periods of time (e.g., hikers, rescue workers in disaster areas, or Third World populations). Harvesting technology may also be relevant for wearable robots and prostheses (e.g., exoskeletons), where the trade-off between battery size and possible operation time is important. For example, Power Knee™, a powered prosthesis with actuation, requires charging after every 6 h [9]. Future wearable robots and prostheses designs inspired by energy harvesting may increase the usage time of these devices.

Biomechanical energy harvesting relies on the fact that the average energy expenditure of an active human (i.e., the energy used by the body) is approximately 1.07*10^7^ J per day [10]. This amount of energy is equivalent to the energy stored in roughly 15 kg of batteries. Furthermore, humans derive energy from food products (e.g., carbohydrates, fats, and proteins), which typically have a specific energy that is 35–100 times higher than that of most currently available batteries [11]. Thus, using the human body to convert the energy stored in food into electrical energy may enable substituting traditional energy sources (e.g., battery, solar panels). In fact, ideally (i.e., ignoring the device mass), even with low total conversion rates (e.g., 10% conversion rate considering muscle and device efficiency), it may be beneficial to replace batteries with food as a source of energy. This is due to the relatively high specific energy of food (vs. batteries).

Four main factors should to be considered when designing biomechanical energy harvesting devices: (1) the electrical power output of the device; (2) whether energy harvesting is achieved unconsciously during natural movement or through intentional activation of the device to create energy (e.g., as in mechanically powered flashlights); (3) convenience of using the device, which can be assessed by factors such as the comfort of the device’s attachment to the human body and the effort exerted to produce the electrical power, measured as metabolic power. Metabolic power refers to the amount of energy required to perform an activity; it is also known as ‘metabolic rate’, and the terms can be used interchangeably; and (4) the advantages and disadvantages of using an alternative power supply unit (e.g., carrying batteries or a solar panel).

Recent research on biomechanical harvesting has focused on increasing harvested electrical power output during walking (while harvesting unconsciously) and on evaluating the metabolic power of generating electrical energy [1,6,7,12]. Niu and colleagues [1] suggest that in order to achieve a lower metabolic power, biomechanical energy harvesting should focus on utilizing the energy dissipating to the surroundings (e.g., energy dissipated in the shoe sole while walking or by the muscle during negative work). In the negative work phase, the muscles perform an eccentric contraction and act as brakes [13]. Thus an external device could partially replace the braking performed by the muscles, and therefore assists with the braking. This is similar to the idea of regenerative braking in a hybrid car, in which power is generated during deceleration. Knee and ankle devices have been built based on this idea [6,14].

Different measures have been implemented to assess the effect of energy harvesting devices on the individuals using them. Rome et al. [7] suggested a measure of the net efficiency of electrical power production: the ratio between power output and metabolic power input. These researchers defined metabolic power input as the difference between walking with the device while harvesting and walking with the device while not harvesting (in the latter case, the device is locked and acts as a regular backpack). Based on this measure, Rome et al. found that the backpack harvester had a 19.5% net efficiency.

Riemer and Shapiro [11] suggested a measure of the overall efficiency, which was defined as:

1 $$ overall\  efficiency=\frac{\Delta electrical\  power}{\Delta metabolic\  power}, $$

where ∆*electrical power* is the electrical power output and Δ*metabolic power* is the difference in metabolic power of a particular activity with and without a device. Based on this measure, the knee device was found to have an efficiency of approximately 7.5%, while the efficiency of the backpack device was approximately 1%.

Donelan et al. [6] proposed measuring the cost of harvesting (COH), defined as:

2 $$ COH=\frac{harvesting\  metabolic\  power\  change}{device\  electrical\  power\  output}, $$

In fact, the COH is the inverse of the net efficiency measure suggested by Rome et al. [7], but in contrast to the net efficiency measure that focuses on the device, the COH focuses on the effect on the user. Based on this formulation, the COH values of the backpack and knee devices [6,7] are approximately 5 and 0.7, respectively. Researchers have attributed the differences in COH of the devices to variations in types of muscle work performed during harvesting. While some devices harvest mainly during positive work (the muscles contract and generate motion, i.e., concentric contraction), others harvest mainly during negative work (the muscles act as brakes to slow down the motion, i.e., eccentric contraction) [13]. Differences in COH of the devices are also attributed to variations in the efficiency of converting mechanical work to electricity.

All the measures described above are useful for evaluating the performance of harvesting devices. Yet, it is still unclear under what conditions would it be beneficial to use an energy harvester instead of batteries to provide electrical power. For example, what energy source should be used by the individual from the opening scenario who is planning a three-week hike?

To answer this question, we introduce a new approach for evaluating biomechanical energy harvesting devices. Our approach is based on the idea that the user should prefer the option that requires the least effort, where effort refers to metabolic power. In line with this approach, we developed a mathematical model that calculates the difference in metabolic power between two states: when using a biomechanical energy harvesting device and when carrying a power supply unit (e.g., a battery or a gasoline generator) that provides the same amount of energy. If the amount of metabolic power required to harvest the electrical energy is lower than the amount necessary to carry the energy source, then biomechanical harvesting is the better option, and vice versa. It is possible to evaluate a specific biomechanical harvester design based on our new approach by experimentally measuring the metabolic power of test subjects walking with a harvester or with a power supply unit. However, when designing a biomechanical harvester, it would be beneficial to evaluate several design concepts without the need fabricate the whole device.

In the next section we present the development of a mathematical model to predict the metabolic difference between using a harvester and carrying a power supply unit, as a function of various parameters. The model development includes a discussion of the relations among parameters and their possible ranges. We then demonstrate two applications of our proposed model. First, we use our model to examine the performance of several of the current energy harvesting devices (case study analysis). Second, we conduct a design scenarios analysis, which assesses how different design parameters would influence the performance of the device and may assist in establishing guidelines for future device development. We conclude with a discussion of the contribution of our research, its limitations, and future research directions.

## The mathematical model

### Overview

In this section we describe the mathematical model and discuss relationships among the parameters included in the model. Our model presents a set of equations, which enables calculating the metabolic power while harvesting energy and while using a power supply unit for given operation conditions. *For simplicity of terms in this paper, hereafter we use the term “battery” to refer to all power supply units (e.g., electrical battery, fuel cells, etc.).*

An operation is defined as a task (e.g., a three-week hike) that requires a certain amount of electrical energy, which can be supplied by either batteries or a harvesting device. Each operation can be characterized by the following parameters: walking speed, walking time, harvested electric power, specific energy of the battery, user’s body mass, COH, device mass, and the location of the device on the human body. The speed of the walking is important, as studies show that the higher the speed, the higher the metabolic power [15,16]. The location of the device on the body is important, as studies show that carrying a mass at more distal locations generally results in higher metabolic power (e.g., [15,17]). In our model, the location on the body can be either the ankle, the knee, or the back.

### Developing the model

Our model allows for cases where not all the energy produced by the harvester is consumed at the time of production. In such cases, it is necessary to include the mass of the battery or capacitor that stores the energy.

Based on our proposed approach, the criterion for determining whether using a biomechanical energy harvester is beneficial can be formulated as follows:

3 $$ Metabolic\  difference= Metabolic\_ powe{r}_{battery}\hbox{--} Metabolic\_ powe{r}_{harvester} $$

where *Metabolic_power*_*battery*_ is the change in metabolic power due to carrying the additional weight of the battery; *Metabolic_power*_*harvester*_ is the metabolic power of using the harvester, which is defined as the difference between walking while harvesting and without the harvesting device.

The metabolic cost of using a battery is a function of the mass of the battery, its location on the body, walking speed, and the human body mass. We assume that the battery mass is always carried on the back. The metabolic power of the battery is therefore:

4 $$ Metabolic\_ powe{r}_{battery} = {f}_1\ \left( battery\_ mass,\ S,\ L\Big|L= back\right)\cdot BM, $$

where *f*_*1*_ calculates the metabolic power of carrying a mass [W/kg], *battery_mass* is the mass [kg] of the battery, *S* is the walking speed [km/h], *L* is the location of the mass on the body (which in our case is assumed to be on the back)*,* and *BM* is the user’s total body mass [kg]. Note that *f*_*1*_ is multiplied by body mass because, typically, metabolic power results and predications are presented as power per kilogram (e.g., [15,16,18,19]). The term *battery_mass* is the mass of a battery that provides the required amount of energy. This can be calculated as follows:

5 $$ battery\_ mass=\frac{3600T\cdot P}{battery\_ specifc\_ energy}, $$

where *T* is the duration of use, indicating the actual time that the harvester is operating (in hours); *P* is the average electrical power harvested [W] by the device at a given walking speed [km/h]; the *battery_specific_energy*, which refers to the carried battery [J/kg].

The metabolic power of the harvester is calculated using the following equation:

6 $$ Metabolic\_ powe{r}_{harvester}={f}_1\left({M}_h,\ S,\ {L}_h\right)\cdot BM+P\cdot COH+{f}_1\left({M}_{hb},\ S,\ {L}_{hb}\right)\cdot BM, $$

where *f*_*1*_ calculates the metabolic power [W/kg] of carrying the harvester device mass *M*_*h*_*,* at walking speed *S,* and on a given body location *L*_*h*_. *COH* is based on equation 2. *M*_*hb*_ is the mass of the harvester battery [kg], and *L*_*hb*_ is its location (e.g., the location can be either on the back or similar to the device’s location).

It is noteworthy that since food-specific energy is high relative to conventional energy sources (35–100 times higher [11]), we ignore the weight of additional food that might be carried by the user to support the extra metabolic cost of harvesting.

We performed a calculation of the $$ Metabolic $$$$ difference $$ for any given scenario, by implementing our model equations using Excel (Microsoft, USA). Additional file 1 presents an example of determining the metabolic power difference using our model.

### Metabolic cost of carrying a mass

Prediction equations are necessary to estimate the metabolic power for carrying a mass *M* at a walking speed *S* on a body location *L*, *f*_*1*_(*M, S, L*). To the best of our knowledge, there are only two studies that provide relevant prediction equations for masses carried on the ankle, knee, or back [19,20]. One study considered conditions of walking on a level treadmill, and provided equations for masses carried on the ankle, knee, or back [20]. Another study considered the terrain factor (e.g., slope, sand), and provided prediction equations for mass carried on the ankle or on the back [19]. In our study we need to know the effect of mass carried at each of the three locations, and therefore we used equations from Schertzer and Riemer [20]. Based on empirical data, the best fit for the metabolic power was obtained using an exponential function [19], with different equations for the various locations (see Table 1). In the current study, the increase in metabolic power due to the addition of mass (device or battery) is calculated by subtracting the metabolic power of walking without a mass from metabolic power of walking with a mass at the given location (see Additional file 1 for an example of these calculations).

**Table 1:** **Prediction equations for metabolic power [W/kg] as a function of speed [km/h] and added mass [kg] for all three body locations** [20]

**Body location**	**Equation**	**R** ^**2**^
Ankle	*e* ^(0.67913 + 0.190769 ⋅ *speed* + 0.075217 ⋅ *mass*)^	0.78
Knee	*e* ^(0.596228 + 0.206819 ⋅ *speed* + 0.059647 ⋅ *mass*)^	0.83
Back	*e* ^(0.518479 + 0.220584 ⋅ *speed* + 0.011237 ⋅ *mass*)^	0.85

Note: For the ankle and knee, the mass refers to the added mass for each leg. Therefore, a mass of 0.5 kg means that the person is carrying 0.5 kg on each leg, a total of 1 kg.

Based on our model, the metabolic power at the different conditions is utilized to calculate the metabolic difference (equation 3). A positive metabolic difference for a given scenario would indicate that the use of an energy harvester is beneficial.

### The model parameters

In this section we examine how to obtain the model parameters, and discuss the relations among the parameters. For example, the body mass and the battery’s specific energy can be determined independently of other parameters. Yet other device parameters, such as mass and electrical power output, are coupled to a certain degree.

#### Power to mass relation

The device’s mass and the electrical power it can supply are coupled to a certain degree. This is due to two main reasons. First, when harvesting energy, the mechanical work derived from human motion is transferred to the device. Therefore, to increase the electrical power, the device should apply larger force/moment, which in turn results in a stronger structure. Second, the size of the energy conversion components is associated with the amount of energy they can provide. For example, with generators based on electrical induction, larger generators will harvest more electrical power. However, power and mass are not fundamentally coupled; it is still possible to change the device mass without changing its electrical power, and vice versa. This is true in cases such as: (1) when using materials with a high strength-to-mass ratio (e.g., replacing aluminum with carbon fiber), (2) when using different harvesting technology (e.g., devices based on electrical induction have a different ratio of mass-to-power than devices based on piezoelectricity), and (3) when using gears with higher efficiency (which will result in higher power for the same mass).

#### Speed and electrical power

For backpack devices, walking at a higher speed will result in higher frequency and oscillation amplitude. Rome et al. [7] found that electrical power increases almost linearly with the speed. For knee and ankle devices, increasing walking speed will result in faster joint angular velocity, which in turn increases the electrical power [21]. Yet no studies empirically investigated this relation in knee and ankle devices. Therefore in our study we do not consider the effect of walking speed on the model results.

#### Cost of harvesting (COH)

As delineated in equation (2), COH is defined by the ratio between the change in metabolic power and the harvested electrical power. COH is affected by several factors. One factor that explains the COH is whether the harvesting is performed during positive or negative muscle power. Based on past research [22], the effect of positive and negative muscle power on the metabolic energy is described as follows:

7 $$ metabolic\  power\left[W\right]=\frac{postive\  power\left[W\right]}{{\ \upeta}^{+}}+\frac{negative\  power\left[W\right]}{{\ \upeta}^{-}}, $$

where η^+^ is the muscle efficiency during positive power, and η^−^ is the muscle efficiency during negative power; positive power efficiency is typically considered to be 25% [22], and negative power efficiency ranges between 28% and 160% [22-25].

It is noteworthy that equation (7) cannot fully explain findings from past studies regarding changes in metabolic power when performing positive and negative power. In line with equation (7), researchers [6,7] showed that their device achieved lower metabolic changes than predicted when assuming that harvesting occurred only during positive muscle power. For example, for a knee device, if all the harvesting would have been performed using positive muscle power, the predicted COH would have been 12.9. Yet the actual COH was 0.7 [3]. This gap was attributed to the fact that the device harvests using primarily negative (rather than positive) muscle power [3]. However, based on published data, we examined the extent to which equation (7) explains the device’s performance. Using the figures from past research [21], we estimated that the devices replaced approximately 6 W of the negative muscle power, and the addition of positive muscle power was approximately 1.4 W. To predict the change in metabolic power, equation (7) was used with an efficiency of 25% for positive work and an efficiency of 100% for negative work. Substituting these values in equation (7) yields a total change in metabolic power of approximately −0.4 W. Yet experimental results from past research indicated that the total change in metabolic power was 5 W [21]. In other words, there is a difference between the predicted and the actual change in metabolic power.

This difference between the predicted and the actual metabolic power suggests that there may be additional underlying mechanisms (e.g., interactions in the muscle tendon unit) that were not taken into account in the model (equation (7)), and which may affect the metabolism. It is also possible that changes in other joints affect the metabolic power, and thus equation (7) should be calculated for all body joints. These observations are in line with recent work [26-28] that examined the interaction of muscle tendon unit work on metabolic changes during hopping, using an ankle exoskeleton. The researchers argued that the assistance of the exoskeleton changed the working point of the muscle (on the force length curve), and that the reduction of metabolic cost could not be explained by the change in net joint work. Therefore, the researchers speculated that perhaps the reduction in metabolic power was due to changes in work performed by other joints.

Another factor that may affect the COH is the amount of harvested electrical power. This is because the harvested electrical power should be lower than the available joint net mechanical power during the negative work phase. It should also be noted that walking on different slopes will change the available joint net mechanical power. On a down slope, for example, the joint performs more negative work than on positive slopes. Further, it is unclear how changes in the ratio of harvested power relative to the available joint power would affect the COH. Thus, more research is needed to predict the COH. We therefore recommend performing an experiment with a prototype that enables changing the harvested power, and then finding the COH as a function of the levels of harvested power for the given design.

## Method

### Case study

To demonstrate the capabilities of our mathematical model, we evaluated three devices: Rome’s backpack harvester [7], Donelan’s knee harvester [6], and an ankle device known as SPaRK (SpringActive Inc., Tempe, AZ, USA) [14]. We do not show calculations for shoe devices (e.g., [2-4]), since there are neither evaluations of the effects on humans nor reports about their masses. However, it is possible to use the formulation for the ankle device to evaluate shoe devices as well.

Our goal in this case study was to find combinations of masses and walking times that would make using the harvesting devices preferable to carrying batteries. To this end, we set the devices’ parameters (e.g., electrical power output, COH, specific energy of batteries) at fixed values, and manipulated the devices’ masses (from 0.1 to 1 kg). For each mass we determined the walking time required in order for the device to become the preferred option over a battery (see Table 2, 3, and 4). Note that the masses of the devices as reported in past studies were 0.75 kg (knee [29]) and 0.86 kg (ankle [30]); the mass for the backpack device was not reported, and hence was estimated to be approximately 1 kg. All these values are in the ranges of the mass explored in the current analysis.

**Table 2:** **Case study for the ankle energy harvesting device** [14]

				**Device mass [kg]**							
		**0.1**	**0.2**	**0.3**	**0.4**	**0.5**	**0.6**	**0.7**	**0.8**	**0.9**	**1**
**Walking time [hours]**	**40**	**−2.0**	**−4.9**	**−7.7**	**−10.6**	**−13.5**	**−16.4**	**−19.3**	**−22.3**	**−25.3**	**−28.3**
	**60**	0.5	**−2.4**	**−5.2**	**−8.1**	**−11.0**	**−13.9**	**−16.8**	**−19.8**	**−22.8**	**−25.8**
	**80**	3.0	0.2	**−2.7**	**−5.6**	**−8.5**	**−11.4**	**−14.3**	**−17.3**	**−20.2**	**−23.2**
	**100**	5.5	2.7	**−0.1**	**−3.0**	**−5.9**	**−8.8**	**−11.8**	**−14.7**	**−17.7**	**−20.7**
	**120**	8.1	5.3	2.4	**−0.5**	**−3.4**	**−6.3**	**−9.2**	**−12.2**	**−15.1**	**−18.1**
	**140**	10.7	7.8	5.0	2.1	**−0.8**	**−3.7**	**−6.6**	**−9.6**	**−12.6**	**−15.6**
	**160**	13.3	10.4	7.6	4.7	1.8	**−1.1**	**−4.0**	**−7.0**	**−10.0**	**−13.0**
	**180**	15.9	13.0	10.2	7.3	4.4	1.5	**−1.4**	**−4.4**	**−7.4**	**−10.4**
	**200**	18.5	15.7	12.8	9.9	7.1	4.1	1.2	**−1.8**	**−4.7**	**−7.7**
	**220**	21.1	18.3	15.5	12.6	9.7	6.8	3.8	*0.9*	***−2.1***	**−5.1**
	**240**	23.8	21.0	18.1	15.3	12.4	9.4	6.5	3.6	0.6	**−2.4**
	**260**	26.5	23.7	20.8	17.9	15.0	12.1	9.2	6.2	3.3	0.3

The values in the table are the differences in metabolic power in watts (carrying batteries minus harvesting device scenarios), for given mass and walking times. Positive values indicate that the harvesting device requires less effort than batteries (i.e., energy harvesting is preferred over batteries), negative values (**bold**) represent combinations were batteries are preferred options. The Italic values refer to the current published device (0.86kg, 220h).

**Table 3:** **Case study for the knee energy harvesting device** [6,29]

					**Device mass [kg]**						
		**0.1**	**0.2**	**0.3**	**0.4**	**0.5**	**0.6**	**0.7**	**0.8**	**0.9**	**1**
**Walking time [hours]**	**40**	**−0.6**	**−2.9**	**−5.1**	**−7.4**	**−9.7**	**−12.0**	**−14.3**	**−16.7**	**−19.0**	**−21.4**
	**50**	1.9	**−0.3**	**−2.6**	**−4.9**	**−7.2**	**−9.5**	**−11.8**	**−14.1**	**−16.5**	**−18.8**
	**60**	4.5	2.3	**0.0**	**−2.3**	**−4.6**	**−6.9**	**−9.2**	**−11.6**	**−13.9**	**−16.3**
	**70**	7.1	4.8	2.6	0.3	**−2.0**	**−4.3**	**−6.6**	**−9.0**	**−11.3**	**−13.7**
	**80**	9.7	7.4	5.2	2.9	0.6	**−1.7**	**−4.0**	**−6.4**	**−8.7**	**−11.1**
	**90**	12.3	10.1	7.8	5.5	3.2	0.9	**−1.4**	**−3.8**	**−6.1**	**−8.5**
	**100**	15.0	12.7	10.4	8.1	5.9	3.5	*1.2*	***−1.1***	**−3.5**	**−5.8**
	**110**	17.6	15.4	13.1	10.8	8.5	6.2	3.9	1.5	**−0.8**	**−3.2**
	**120**	20.3	18.0	15.8	13.5	11.2	8.9	6.5	4.2	1.9	**−0.5**
	**130**	23.0	20.7	18.4	16.2	13.9	11.6	9.2	6.9	4.5	2.2

The values in the table are the differences in metabolic power in watts (carrying batteries minus harvesting device scenarios), for given mass and walking times. Positive values indicate that the harvesting device requires less effort than batteries (i.e., energy harvesting is preferred over batteries), negative values (**bold**) represent combinations were batteries are preferred options. The Italic values refer to the current published device (0.75kg, 100h).

**Table 4:** **Case study for the backpack energy harvesting device** [7]

					**Device Mass [kg]**						
		**0.1**	**0.2**	**0.3**	**0.4**	**0.5**	**0.6**	**0.7**	**0.8**	**0.9**	**1**
**Walking time [hours]**	**170**	**−0.1**	**−0.6**	**−1.2**	**−1.8**	**−2.4**	**−3.0**	**−3.6**	**−4.2**	**−4.8**	**−5.4**
	**180**	0.6	0.0	**−0.6**	**−1.2**	**−1.8**	**−2.4**	**−3.0**	**−3.6**	**−4.2**	**−4.8**
	**190**	1.2	0.6	0.0	**−0.6**	**−1.2**	**−1.8**	**−2.4**	**−3.0**	**−3.6**	**−4.2**
	**200**	1.8	1.2	0.6	0.0	**−0.6**	**−1.2**	**−1.8**	**−2.4**	**−3.0**	**−3.6**
	**210**	2.4	1.8	1.2	0.6	0.0	**−0.6**	**−1.2**	**−1.8**	**−2.4**	**−3.0**
	**220**	3.0	2.4	1.8	1.2	0.6	0.0	**−0.6**	**−1.2**	**−1.8**	**−2.4**
	**230**	3.6	3.0	2.4	1.8	1.2	0.6	0.0	**−0.6**	**−1.2**	**−1.8**
	**240**	4.2	3.6	3.0	2.4	1.8	1.2	0.6	0.0	**−0.6**	**−1.2**
	**250**	4.8	4.2	3.6	3.0	2.4	1.8	1.2	0.6	0.0	**−0.6**
	**260**	5.4	4.8	4.2	3.6	3.0	2.4	1.8	1.2	0.6	*0.0*

The values in the table are the differences in metabolic power in watts (carrying batteries minus harvesting device scenarios), for given mass and walking times. Positive values indicate that the harvesting device requires less effort than batteries (i.e., energy harvesting is preferred over batteries), negative values (**bold**) represent combinations were batteries are preferred options The Italic value refers to the current published device (1kg, 260h).

#### Values in the case study analysis

Our analysis considered the following values: In all three cases, the specific energy of the battery was set to 7.2*10^5^ J/kg; this value represents the higher end of current lithium-ion batteries [31]. In all three cases, the human mass was set to 75 kg. The manipulated mass of the devices ranged from 0.1 to 1 kg, with intervals of 0.1 kg. It is noteworthy that the energy harvesting device includes a storage battery that can store the produced energy if this harvested energy is not used immediately. The size of the storage battery depends on the amount of energy the user wishes to store. In the current analysis, we assumed that a small storage battery mass is needed, and therefore we included it in the device’s mass.

#### Device-specific parameters

Device parameters were extracted from published data, when such data was available; when data was not available, engineering judgment was used to estimate the parameters.

*Ankle device*. COH value for the ankle device was not reported in the literature. Several factors may affect the COH of ankle devices. The SPaRK ankle device was designed to harvest only during the negative muscle work. Yet a high proportion of the negative work at the ankle joint is accounted for by elastic storage of energy, which is later returned during positive work. Consequently, harvesting during negative work may be problematic. On the other hand, recent work shows that the metabolic cost of statically holding the muscle during elastic storage is high, and therefore there is no metabolic benefit from using elastic storage [32]. Thus, reducing load on the muscle during stretching may reduce the metabolic cost. Due to these contradictory findings, we assumed a COH value of 0.7, which is equal to the knee device. The electrical power output was 6 W (assuming there were two devices and an output of 3 W per ankle) for a walking speed of 4.83 km/h [30].

*Knee device*. Based on [6,29], COH for this device is 0.7, and the electrical power output is 12 W (assuming two devices and an output of 6 W per knee) for a walking speed of 5 km/h.

*Backpack device*. COH was 4.8, and the harvested electric power was 2 W. The walking speed was 5 km/h, and the backpack mass (including the device itself) was 29 kg [7]. A more recent study of the same device reports a higher electrical power output of 16 W with a 36 kg backpack [12]. However, this value was achieved with a device that simulated human motion, rather than an actual human. Therefore, the effect of the harvesting on the metabolic cost is unknown. In the equation for the metabolic cost of carrying a mass at a given speed we used 29 kg as a baseline for the backpack weight, and the device or battery mass was added to this value.

### Design scenarios analysis

We performed a design scenarios analysis to examine how future changes in the energy harvesting devices’ parameters could affect the performance of the devices. Using our proposed model, we determined how combinations of four design parameters – battery specific energy, device mass, harvested electrical power output, and COH – would affect the amount of walking time necessary for the harvester to become the preferred option over batteries. We assumed that all these parameters were not coupled (e.g., we could increase harvested electrical power without any change in the device mass).

The analysis was performed for nine scenarios, each of which assumed a human weighing 75 kg, walking at 5 km/h. The scenarios are described in Table 5. They varied in the following main factors: Seven scenarios considered specific energy that is equal to current available batteries (7.2*10^5^ J/kg; scenarios A-G), and two scenarios considered a future improvement of 50% over current batteries (scenarios H and I; 10.8*10^5^ J/kg). The device mass ranged from 0.1 to 0.5 kg, with 0.1-0.2 kg considered as low weight (scenarios A, B, C, D, and H), and 0.3-0.5 kg considered as high weight (scenarios E, F, G, and I). Note that for the ankle and knee devices, the mass value is the mass of one device at each location (e.g., 0.1 kg on the left ankle).

**Table 5:** **Design scenarios analysis**

		**Design scenarios options**									
		**Scenario A**	**Scenario B**	**Scenario C**	**Scenario D**	**Scenario E**	**Scenario F**	**Scenario G**	**Scenario H**	**Scenario I**	**Current devices**
**Scenario description**	Specific energy	7.2*10^5^							10.8*10^5^ (50% better than current batteries)		7.2*10^5^
		(similar to current available batteries)									
	Device mass	Low				High			Low	High	Device specifications are based on published data
	Power output	Low				High			Low	High	
**Ankle device**	Device mass	0.1	0.2	0.2	0.1	0.3	0.5	0.5	0.1	0.5	0.86
	COH	0	0	−1	−1	0	−1	0	−1	−1	0.7
	Power output	4	4	4	3	15	10	15	3	10	6
	***Walking time (results)***	***34***	***68***	***21***	***0***	***27***	***22***	***45***	***0***	***33***	***224***
**Knee device**	Device mass	0.1	0.2	0.2	0.1	0.3	0.5	0.5	0.1	0.5	0.75
	COH	0	0	−1	−1	0	−1	0	−1	−1	0.7
	Power output	4	4	4	3	15	10	15	3	10	12
	***Walking time (results)***	***27***	***54***	***7***	***0***	***22***	***7***	***36***	***0***	***11***	***98***
**Backpack device**	Device mass	0.1	0.2	0.2	0.1	0.3	0.5	0.5	0.1	0.5	1
	COH	0	0	1	1	1	0	0	0	0	4.8
	Power output	4	4	4	4	15	10	15	4	10	2
	***Walking time (results)***	***5***	***10***	***44***	***39***	***37***	***10***	***7***	***7***	***37***	***260***

The electrical power output ranged between 3 and 15 W, with power output in the range of 3-4 W considered low (scenarios A, B, C, D, and H) and 10-15 W considered high (scenarios E, F, G, and I). For the ankle and knee devices, power output was the sum of two devices (i.e., one for each leg); for the backpack device power output was based on one device. The ranges were chosen based on the total negative work performed at each location during level walking. For the ankle and knee devices, negative muscle power is approximately 20 W and 33 W, respectively, for an 80 kg subject [33]. For the backpack device, the estimate is approximately 20 W [11]. The maximum energy that can be harvested was set to be lower than these values (15 W). This is due to conversion losses and to limitation on the maximum energy that could be harvested without interfering with the walking pattern.

The COH values for the ankle and knee devices were either −1 or 0, and for the backpack device were either 0 or 1. The COH values, which rely on findings from literature (e.g.,[6]), are somewhat arbitrary, since it is unclear what determines this parameter (see the Model Parameters section). Furthermore, research shows that even for the same device COH values vary significantly from person to person. For example, the COH of the knee device ranged from −6.5 to 6 and for the backpack from −0.47 to 7.8 (both experiments involved six test subjects [6,7]).

Our design scenarios analysis involved the calculation of the required walking time for nine combinations of parameters. For the ankle and knee devices these nine combinations were similar; for the backpack a different set of parameters was used (see Table 5).

## Results

### Case study results

Results of the case studies for the ankle, knee, and backpack devices appear in Tables 2, 3, and 4, respectively. The results show that the ankle energy harvesting device with the examined parameters (as described in the Method section) would require approximately 227 h of walking to become the preferred option over batteries. In this case, the battery mass would be 6.81 kg.

For the knee device with the examined parameters, results show that the harvesting device would be the preferred option over batteries for walking times longer than 98 h. For this walking time, the battery mass would be 5.88 kg.

The backpack device with the examined parameters would become the preferred option over batteries for walking times longer than 260 h. For this walking time the battery mass would be 2.6 kg.

#### Results of the design scenarios analysis

The design scenarios analysis reveals that the ankle energy harvesting device would be preferred over batteries for walking times shorter than 25 hours, but only for parameter combinations where the COH was −1 (i.e., Ankle options C, D, F, and H in Table 5). Further, the analysis shows that for low electrical power and low masses with COH of −1, the device was not sensitive to changes in the specific energy (see Table 5, ankle combinations D vs. H).

For the knee device, the design scenarios analysis reveals that the harvesting device would be preferred over batteries for walking times shorter than 25 hours for parameter combinations C, D, E, F, H and I. Under all of these parameter combinations except one (combination E), the COH was −1. Further, the combinations with COH of −1 were not sensitive to changes in the specific energy (i.e., scenarios D vs. H and F vs. I).

Finally, the backpack device would be preferred over batteries for walking times shorter than 25 hours for parameter combinations A, B, F, G, and H. Under all of these parameter combinations the COH was 0. Here again parameter combinations with low COH (COH = 0) and low masses were not sensitive to changes in specific energy (A vs. H).

## Discussion

### Contribution

This paper presents a new approach for evaluating energy harvesting devices. The proposed approach compares the metabolic power while harvesting energy with the metabolic power while carrying batteries, given that both options provide equal amounts of energy. In order for the harvesting device to be preferred over batteries, the device’s design parameters should indicate a lower metabolic power for generating energy using the harvester than when carrying batteries.

Our model enables an assessment of the difference in metabolic power between the two options, for a given set of design parameters. Thus, it may be useful in determining the parameters for improving the device’s performance, and therefore offers device designers a tool that can save the time and expenses involved in developing and evaluating harvesting devices.

Our case study analyses of current devices revealed that to become the preferred option, the ankle device (electrical power output 6 W) would require approximately 227 h of walking, the knee device (12 W) 98 h of walking, and the back device (2 W) 260 h of walking. In practical terms, this means that these devices are not close to becoming an alternative to batteries for a three-day hike. However, for a three-week trek in a remote part of the world, they may be relevant.

The design scenarios analysis suggests that future technological development should be focused on light devices with low COH, even if these devices harvest less than 5 W. The fact that devices with low masses, low electric power, and low COH could become better than batteries in a relatively short walking time is encouraging, since it is easier to build devices with such specifications than devices with high power and low masses (as was suggested in recent development attempts, e.g., [6,7]).

It is generally agreed that the metabolic power, and thus the COH as well, are associated with to the type of muscle work involved during the device’s operation [6,7,34]. Yet it is still unclear which parameters should be changed to reduce the COH (e.g., timing or magnitude of harvesting). Moreover, it is known that other factors, such as muscle force production and force production rate, can affect metabolic power [35-38]. More research in this area is necessary to predict how device design parameters would affect COH.

Future development in battery technology may have an influence on harvesting device development. The rule of thumb regarding batteries is that their specific energy doubles every decade [4]. In the long run, a continual increase in this factor (i.e., higher specific energy) may lead to an increase in the required walking time for harvesting devices to be the preferred option. Yet the design scenarios analysis suggests that devices with a low mass and low COH can still be beneficial, even with a 50% increase in the batteries’ specific energy as compared to current batteries (i.e., with future specific energy of 10.8*10^5^ J/kg).

With some modifications the approach and model proposed in this research could be used to evaluate other energy solutions, such as solar panels and gasoline generators. An analysis of a few commercial products (Additional file 2) shows that after 13 hours of use, a solar panel providing 7 W and weighing 0.46 kg would become a better source of energy than a battery. This calculation, however, is based on the assumption that there is full exposure to the sun (i.e., no clouds or trees) and that the panel is correctly oriented towards the sun. Without these conditions, solar panel performance significantly deteriorates (as much as 10% lower than nominal values [8]). A gasoline generator providing 900 W for 3.8 hours with a weight of 14.9 kg (including the gasoline) would be equivalent to a battery mass of 17.1 kg.

Our approach can also address scenarios that were not analyzed in this paper, such as selective use of an energy harvester. For example, a user might carry the device on his/her back while walking uphill, but use it only while walking downhill where there is more negative work. In such cases, it would be necessary to slightly modify the mathematical formulation. Instead of comparing the metabolic power for each option (harvesting vs. battery), the total metabolic energy in each option should be considered, as follows: (1) calculate the total additional energy required for carrying the harvesting device uphill, (2) calculate the total change in metabolic energy while walking downhill, (3) calculate the battery mass using the total electrical energy harvested while walking downhill divided by the specific energy of the battery (equation 5), and (4) calculate the cost for carrying the battery uphill and downhill (for a given mass). Note that since the prediction equations for metabolic power as a function of the added mass are for a given walking speed, it is possible to calculate the total change in metabolic energy by calculating the required walking time multiplied by the metabolic power.

### Limitations and future directions

Several limitations in this paper, which offer opportunities for future research, should be acknowledged. First, our analyses were performed on level walking surfaces using equations based on treadmill walking. Past research showed that treadmill walking is similar to walking over ground [39,40]. However, an actual device would typically be used for walking over different terrains and slopes, and thus having data for such conditions would be useful. We know of only one study that provides equations for the cost of carrying a mass on the ankle and back while walking on different terrains and slopes [19]. The prediction equation from that study [19] could be used with our mathematical model to examine ankle and back devices used while walking on different terrains and slopes. This can be done by replacing the metabolic power equations for carrying a mass in our model with those presented in [19]. For knee devices, no studies provide equations to predict the metabolic power during walking on slopes and different terrains. Future work is needed to develop these equations.

Second, we assumed that for the ankle and knee devices the user does not carry a backpack. Several studies indicated that the increase in the metabolic power of carrying a mass on the back is similar to an increase in body mass [20,41-43]. Thus, to predict metabolic changes in cases where the user does carry a backpack while using an ankle or knee device, it is possible to regard the mass on the back as added body mass. In addition, it is likely that a person walking with a heavier load will have larger torque, and therefore more power would be available for harvesting.

Third, our mathematical model is based on regression equations, which are specific to average populations and to specific tasks (i.e., walking). Additional factors that have not been included in the model, such as fitness level, have not been explicitly modeled. Future research should extend our model to include such factors.

Finally, it is noteworthy that research shows that changes in metabolic power while carrying a load are higher for heavier (vs. lighter) individuals (e.g., [15,19,20]. Results of the analysis, therefore, depend on the individual mass (see equations (4) and (6)). This should be taken into account when designing for different individuals.

## Conclusion

This paper introduces a new evaluation approach for energy harvesting devices. The proposed mathematical model enables assessment of energy harvesting devices based on their design parameters. Our analyses reveal that current ankle, knee, and back devices would require a long walking time to become the preferred option over batteries. In order for harvesting devices to be preferred over batteries for shorter walking times, future improvements should focus on reducing the devices’ masses and COH.

## Additional files

Additional file 1:
**An example of determining the metabolic power difference.**
Additional file 2:
**An analysis of a few commercial products.**

## Abbreviations

*COH*: Cost of harvesting
*Metabolic_power*_*battery*_: Metabolic power of carying batteries
*Metabolic_power*_*harvester*_: User Metabolic power of using harvester
*M*_h_: Harvesting device mass
*M*_hb_: Harvesting device battery mass
*S*: Walking speed
*L*_*h*_: Location of the harvester mass on the body
*L*_*hb*_: Location of the harvester battery mass on the body (e.g., the location can be the same as that of the device or it can be a location on the back)
*BM*: User’s total body mass
*P*: Total electrical power harvested
*battery_mass*: The mass of a battery
*T*: Time in hours
*battery*: Specific energy
η^+^ η^−^: The muscle efficiency at positive and negative work
