# Supplementary material for: Harvesting biomechanical energy or carrying batteries? An evaluation method based on a comparison of metabolic power
Source: J Neuroeng Rehabil. 2015 Mar 20;12:30. doi: 10.1186/s12984-015-0023-7 (PMC4375935; doi:10.1186/s12984-015-0023-7)
Supplement: Additional file 1: — An example of determining the metabolic power difference. [file 12984_2015_23_MOESM1_ESM.docx]

**Appendix** **1** : an example of determining the metabolic power difference.

In this appendix we demonstrate the use of our model to assess a device with a given set of design parameters. In this example, we predict the difference in metabolic power between using an energy harvesting ankle device and carrying batteries. We assume that the energy storage component of the device is relatively small, and that it is part of the device’s mass (and on the same location).

The design parameters are: walking speed 5km/h; electrical power output for each leg is 3W (total 6W); COH=0.7 per mass of 0.8 kg per leg; and a user mass of 75kg. The calculation is based on walking time of 100 hours.

We assumed that the battery is carried on the back, and therefore the equation for the metabolic power for carrying the battery has the following form:

$$Metaboli{c power}_{battery}= =\left[ e^{\left( 0.518479+0.220584\cdot speed+0.011237\cdot battery\_mass \right)}-e^{\left( 0.518479+0.220584\cdot speed \right)} \right]\cdot BM$$

Where *battery_mass* is the mass of a battery that provides the required amount of energy, *BM*- body mass

To calculate the battery mass, we used equation (5)

$battery mass=\frac{3600\cdot100\cdot6}{7.2\cdot{10}^{5}}=3kg$

Then, the metabolic power for carrying the batteries is:

$$Metaboli{c power}_{battery}=$$

$$=\left[ e^{\left( 0.518479+0.220584\cdot5+0.011237\cdot3 \right)}-e^{\left( 0.518479+0.220584\cdot5 \right)} \right]\cdot75=12.5 W$$

To calculate the metabolic power for using the energy harvester, we used the following equation:

$Metaboli{c power}_{harvester}=$


$$=\left[ e^{\left( 0.679+0.191\cdot speed+0.075\cdot M_{h} \right)}-e^{\left( 0.679+0.191\cdot speed \right)} \right]\cdot BM+P\cdot COH$$

where *M_h_* is harvesting device mass. Substituting the design parameters, enables calculating the change in metabolic power due to the use of the harvester:

$Metaboli{c power}_{harvester}=$


$$=\left[ e^{\left( 0.679+0.1908\cdot5+0.075\cdot0.8 \right)}-e^{\left( 0.679+0.191\cdot5 \right)} \right]\cdot75+6\cdot0.7=27.3 W$$

𝑀𝑒𝑡𝑎𝑏𝑜𝑙𝑖𝑐 𝑑𝑖𝑓𝑓𝑒𝑟𝑒𝑛𝑐𝑒 =

$=Metaboli{c power}_{battery}-Metaboli{c power}_{harvester}$*=12.5-27.3=-14.7 W*

This indicates that for the given design parameters, with a walking time of 100 hours, it is better to carry a battery than to use the energy harvester.
