# Supplementary material for: Harvesting biomechanical energy or carrying batteries? An evaluation method based on a comparison of metabolic power
Source: J Neuroeng Rehabil. 2015 Mar 20;12:30. doi: 10.1186/s12984-015-0023-7 (PMC4375935; doi:10.1186/s12984-015-0023-7)
Supplement: Additional file 2: — An analysis of a few commercial products. [file 12984_2015_23_MOESM2_ESM.docx]

**Appendix 2:** An analysis of a few commercial products

In this appendix we compare the energy provided by batteries to the energy supplied by two alternative energy sources – a gasoline generator and solar panels. Consistent with the approach suggested in this paper, here again the idea is to choose the alternative that is easier to use. In the cases of these alternatives, the only cost (metabolic power) to the human body is carrying the mass of the device or the battery. Assuming that both alternatives will be carried on the same body location, one needs to compare the mass of the two alternatives providing the same amount of electrical energy, and to choose the lighter alternative.

**Gasoline generator (**Honda GXH50 EU1000i)[1]

Specifications:

- Power output: 900w rated
- Fuel tank capacity: 0.6 gal
- Dry weight: 29 lbs
- Run time at full load: 3.8 h@ full load

To compare the gasoline generator to the battery, one needs to determine the total energy that can be provided by the generator, and then calculate the mass of a battery that provides similar amount of energy.

*Total mass= dry mass+ fuel mass=*13.2 +1.7 =14.9 *kg*

$$Total energy= rating power\cdot working time= 900 W \cdot3.8 hours$$

$Total energy= 900 W \cdot3.8\cdot3600=12312000J$

Equivalent battery mass:

$$Total mass=\frac{total energy}{specific energy}=\frac{12312000}{7.2\cdot{10}^{5}}=17.1kg$$

This suggests that the gasoline generator is the better option, since it has a lighter mass. Note there are many gasoline generators on the market that could be evaluated, and some may perform better than the one selected for this analysis.

**Solar panel (**Goal Zero Nomad 7 solar panel) [2]

We next tested how a solar panel compares to a battery.

Specifications:

- Power output: 7w
- Weight: 0.46 kg
- Cell Type: Monocrystalline
- Dimensions (unfolded): 9 x 1.5 x 17 in (22.9 x 3.8 x 43.2 cm)

In this case the longer the solar panel is used, the more energy is stored. Therefore, we calculated how many working hours are required for the solar panel to become the preferred option by calculating the energy that could be stored in a battery with the same mass.

$$H=\frac{energy that can be stored in battery}{energy that the solar panel produce in hour}$$

$$=\frac{0.46\cdot7.2\cdot{10}^{5}}{7\cdot3600}=13.14 hours$$

The results indicate that when the solar panel works for 13.14 hour and produces an average of 7W during this time period, it becomes a better option compared to the battery.

Note that solar panels have their limitations. For example, if the panel is not positioned in the right orientation with regard to the sun, there might be as much as a 90% drop in the energy it produces. In addition, the panel will not generate the same amount of energy when the user is walking in the woods or in a shaded area [3].

**References**

1. <http://www.electricgeneratorsdirect.com/manuals/EU%20Series%20Specs.pdf>
2. <http://www.goalzero.com/solar-panels>

3. Rome LC, Flynn L, Goldman EM, Yoo TD: Generating electricity while walking with loads. Science, New York, NY 2005, 309:1725-1728
